# Hydrogel/β-FeOOH-Coated Poly(vinylidene fluoride) Membranes with Superhydrophilicity/Underwater Superoleophobicity Facilely Fabricated via an Aqueous Approach for Multifunctional Applications

**DOI:** 10.3390/polym15040839

**Published:** 2023-02-08

**Authors:** Yin Tang, Tang Zhu, Huichao Liu, Zheng Tang, Xingwen Kuang, Yongna Qiao, Hao Zhang, Caizhen Zhu

**Affiliations:** 1 Institute of Low-Dimensional Materials Genome Initiative, College of Chemistry and Environmental Engineering, Shenzhen University, Shenzhen 518060, China; 2 College of Textile Science and Engineering (International Institute of Silk), Zhejiang Sci-Tech University, Hangzhou 310018, China

**Keywords:** hydrogel coating, photo-fenton catalysis, PVDF membrane, oil/water separation, water purification

## Abstract

Hydrogel coatings that can endow various substrates with superior properties (e.g., biocompatibility, hydrophilicity, and lubricity) have wide applications in the fields of oil/water separation, antifouling, anti-bioadhesion, etc. Currently, the engineering of multifunctional hydrogel-coated materials with superwettability and water purification property using a simple and sustainable strategy is still largely uninvestigated but has a beneficial effect on the world. Herein, we successfully prepared poly(2-acrylamido-2-methyl-1-propanesulfonic acid) hydrogel/β-FeOOH-coated poly(vinylidene fluoride) (PVDF/PAMPS/β-FeOOH) membrane through free-radical polymerization and the in situ mineralization process. In this work, owing to the combination of hydrophilic PAMPS hydrogel coating and β-FeOOH nanorods anchored onto PVDF membrane, the resultant PVDF/PAMPS/β-FeOOH membrane achieved outstanding superhydrophilicity/underwater superoleophobicity. Moreover, the membrane not only effectively separated surfactant-stabilized oil/water emulsions, but also possessed a long-term use capacity. In addition, excellent photocatalytic activity against organic pollutants was demonstrated so that the PVDF/PAMPS/β-FeOOH membrane could be utilized to deal with wastewater. It is envisioned that these hydrogel/β-FeOOH-coated PVDF membranes have versatile applications in the fields of oil/water separation and wastewater purification.

## 1. Introduction

Nowadays, the increasing oily wastewater originating from oil spills, industrial chemicals, and daily life oil usage has resulted in detrimental environmental issues as well as the contamination of water resources [1]. To address this challenge, there is an urgent demand for developing endurable, efficient, environmentally friendly and sustainable materials and techniques that can effectively purify oily wastewater. Recently, membrane materials with high separation efficiency, low-cost, energy-efficiency, and flexibility have definite advantages over most traditional approaches (including centrifugation, oil skimmers, magnetic separations, etc.) to purify oily wastewater [2,3,4,5]. During the separation process, however, most of the conventional polymeric filtration membranes with hydrophobic/oleophilic property, such as polypropylene (PP), polytetrafluoroethylene (PTFE), and poly(vinylidene fluoride) (PVDF) membranes, suffer from membrane fouling and pore blocking, thus resulting in a rapid decline of separation performance in practical applications [6,7]. Therefore, a facile, inexpensive, and eco-friendly applicable approach towards modifying the polymeric filtration membrane surface from hydrophobicity to superhydrophilicity/underwater superoleophobicity is highly desired. 

Currently, combining hierarchical microstructures and high surface energy to design and create a superhydrophilic/underwater superoleophobic surface, membrane materials with such special wettability have been used for effectively removing oil from oily wastewater [8,9,10,11,12,13]. Especially hydrogel coatings, possessing crosslinked polymer networks filled with abundant water, have created enormous interest in designing and constructing underwater superoleophobic materials [14,15,16,17]. For instance, Teng et al., successfully prepared the perforated poly(N-isopropylacrylamide)-clay nanocomposite hydrogel, which exhibited outstanding underwater superoleophobicity, high mechanical property, and efficient oil/water separation capability [18]. Matsubayashi et al., reported a fabrication method for calcium alginate hydrogel-coated meshes and fabrics, which could be capable of separation of oil/water mixtures driven by gravity even in various hash environments [19]. Xie and co-workers successfully fabricated cellulose hydrogels with excellent compressive strength via physical dissolution/regeneration process [20]. Moreover, the as-prepared cellulose hydrogel could be utilized as coatings on stainless meshes to separate oil/water mixtures with high efficiency by gravity. However, such hydrogel-coated membranes fail to remove insoluble oils and soluble dyes in a complex sewage system, which limits their practical use extremely. Even worse, these membranes may suffer from secondary pollution caused by the absorption of dyes on membrane surface during the separation process. Therefore, to address the above issues, a rational design of superwetting membranes that can simultaneously remove insoluble oils and soluble organic dyes is highly required for practical wastewater treatment. 

In recent years, advanced oxidation processes (AOPs) with a striking feature of strongly oxidizing radicals have emerged as a promising strategy for the destruction of various organic pollutants without selectivity [21,22,23,24,25]. Among AOPs, semiconductor photocatalysis, a green and sustainable technology, has attracted increasing attention as an efficient tool for degrading organic contaminants and purifying wastewater [26,27]. The transition-metal oxides such as V_2_O_5_, ZrO_2_, WO_3_, and TiO_2_ have been widely applied to water remediation under visible or UV light irradiation [28,29,30,31,32]. Besides semiconductor photocatalysis, photo-Fenton reaction is another universal and powerful tool to eliminate organic contaminants in wastewater [33,34,35,36]. In combination of the advantages of both Fenton reactions and photocatalysis, the photocatalysis-Fenton system (photo-Fenton) may significantly enhance the purification ability. Especially β-FeOOH, an encouraging photo-Fenton catalyst with a narrow bandgap of about 2.06 eV, has been extensively used to decompose organic pollutants under visible light irradiation owing to its low cost and facile preparation [37,38]. Very currently, the photo-Fenton process integrating with membrane technology has been proven to remarkably improve separation performance and self-cleaning ability of the membrane. For example, Wang et al., reported a novel strategy to prepare β-FeOOH mineralized poly(ethylene terephthalate) (PET) fabric through radiation-induced graft polymerization (RIGP) and an in situ mineralization process [39]. More importantly, the resultant PET fabric featured efficient oil/water separation, outstanding visible-light photocatalytic ability, and excellent durability. Xie et al., have successfully fabricated photo-Fenton self-cleaning membranes via green tannic acid (TA)-Fe(III) complexes and mineralization process of β-FeOOH [40]. Furthermore, the as-obtained membranes exhibited high efficiency for various oil/water emulsions and robust photo-Fenton catalytic activity, which demonstrated the removal of oils and organic pollutants simultaneously. Zhang et al., presented a facile method to prepare stabilized polyacrylonitrile (SPAN) nanofiber/β-FeOOH composite membranes, which achieved superhydrophilicity and underwater superoleophobicity [41]. In addition, the as-fabricated membranes could effectively separate oil/water mixtures, remove soluble dyes, and present outstanding stability even in hash conditions. Therefore, combining membrane separation and photocatalysis was proved a useful way for dealing with complex wastewater systems. To our knowledge, hydrogel-coated membranes with photocatalytic activity for oil/water separation and water purifying have not been reported until now. 

Herein, multifunctional PVDF membranes possessing excellent superhydrophilicity, underwater superoleophobicity, and photocatalytic ability are achieved by constructing PAMPS hydrogel/β-FeOOH nanorods composites on the membrane, which have a micro/nano hierarchical structure on the surface. Importantly, the as-prepared hydrogel/β-FeOOH-coated PVDF membranes can effectively remove insoluble oils from the complex wastewater system and can be reusable without losing the separation efficiency. Compared with the previously reported underwater superoleophobic PVDF membranes, the membranes presented in this work simultaneously decompose soluble dyes to realize water purification under visible-light radiation. We envision this simple aqueous strategy to be extended to other polymeric membranes with versatile applications in the field of oil/water separation and where water purification is highly desired.

## 2. Materials and Methods

### 2.1. Material

Poly(vinylidene fluoride) (PVDF) membranes (pore size: 0.22 μm, diameter: 50 mm) were obtained from Alibaba Co., Ltd., 2-Acrylamido-2-methylpropane sulfonic acid (AMPS), ammonium persulphate (APS), poly(ethylene glycol) diacrylate (PEGDA-400, M_n_ = 400 g/mol), and polyacrylamide (PAM, M_n_ = 500 × 10^4^ g/mol) were obtained from Macklin Biochemical Co., Ltd., (Shanghai, China). Toluene, dichloroethane, petroleum ether, hexane, hexadecane, sodium dodecyl sulfate (SDS), iron chloride hexahydrate (FeCl_3_·6H_2_O), and methylene blue (MB) were provided by the Aladdin Chemistry Co. Ltd. (Shanghai, China). All the reagents were used as received in our experiment without further treatment.

### 2.2. Preparation of Hydrogel-Coated PVDF Membranes

The hydrogel-coated PVDF membranes were prepared using a free radical polymerization process. In a typical process, 12.0 g AMPS was added into deionized water, followed by 0.12 g APS, and the mixture was stirred for 10 min. Next, 0.12 g PEGDA-400 as the cross-linker and 0.08 g PAM as the thickener were added, then stirred for 3 h to form a homogeneous solution. A piece of PVDF membrane pre-wetted by ethanol was immersed in the mixed solution. Subsequently, the PVDF membrane covered with sticky solution was taken out after 10 min and placed in an oven at 60 °C for 10 h. The resulting PAMPS hydrogel-coated PVDF membranes were denoted as PVDF/PAMPS_x_ (x represents the AMPS/H_2_O weigh ratio) and stored in pure water for further usage.

### 2.3. Fabrication of Hydrogel/β-FeOOH-Coated PVDF Membranes

The hydrogel/β-FeOOH-coated PVDF membranes were obtained by a simple in situ mineralization process. In brief, a piece of PVDF/PAMPS membrane was immersed in 40 mL of FeCl_3_·6H_2_O aqueous solution (concentration from 2 to 8 mg/mL). Then, 20 mL of HCl (0.01 M) aqueous solution was added into the above FeCl_3_ solution under stirring, and the mixed solution was incubated at 60 °C for 24 h. The resulting hydrogel/β-FeOOH composite membranes were obtained after rinsing with deionized water and stored in pure water for further experiment. The as-obtained membranes were referred as PVDF/PAMPS/β-FeOOH_y_, where y refers to the mass concentration of FeCl_3_·6H_2_O aqueous solution.

### 2.4. Preparation and Separation of Oil-In-Water Emulsion

In this work, an oil-in-water emulsion was prepared by mixing 98.0 g H_2_O and 2.0 g hexadecane with the addition of 10 mg SDS and stirring at 6000 rpm for 6 h at room temperature. The PVDF/PAMPS/β-FeOOH membranes stored in deionized water before separation was placed on a filtration device. Then, the translucent emulsion (20 mL) was poured onto the apparatus and the diameter of the PVDF/PAMPS/β-FeOOH membrane was 16 mm during the separation process. The separation process was conducted under a constant pressure of 0.1 MPa. The separation efficiency (R, %) was calculated by the following equation [42]:

(1) $$R=\left(1-\frac{C_{f}}{C_{0}}\right)\times 100\%$$

where *C_f_* and *C*_0_ are the oil concentration in the filtrate and the feed solution, respectively.

### 2.5. Evaluation of the Photo-Fenton Activity

The photo-Fenton performance of the as-prepared PVDF/PAMPS/β-FeOOH membranes was evaluated by MB degradation. A Xenon lamp (300 W) with a 420 nm optical filter (PLS-SXE300/300UV, Perfect-Light, China) was utilized as a typical visible-light source. Before irradiation, the as-fabricated membrane (diameter: 50 mm) was immersed into a mixed MB aqueous solution (10 mg/L, 50 mL) with H_2_O_2_ (10 mmol, 50 μL), and stored for 30 min in the dark to reach the adsorption equilibrium. After a certain time of visible light irradiation, 2 mL of the above solution was taken out to measure the residual MB concentration by a UV-vis spectrophotometer (UV-1800, Shimadzu, Kyoto, Japan).

### 2.6. Characterization of Hydrogel/β-FeOOH Composite Membranes

Scanning electron microscopy (SEM, JSM-7800F, JEOL Ltd., Tokyo, Japan) was conducted to elucidate the morphology of the membranes. The crystal structure of the as-obtained membranes was investigated by an X-ray diffractometer (MiniFlex 600, Rigaku, Tokyo, Japan) with a Cu-Kα radiation from 5° to 90° at a rate of 5° min^−1^. Attenuated total reflection infrared spectrometry (ATR-IR, Bruker Tensor 27, Bruker Optics GmbH, Ettlingen, Germany) and X-ray photoelectron spectroscopy (XPS, Escalab-250Xi, Thermo Scientific, Waltham, MA, USA) were used to analyze the surface chemical compositions of the membranes. Water contact angle (WCA) and underwater oil contact angle (UOCA) were measured by a contact angle meter (SDC-350, SINDIN, Dongguan, China) at room temperature. The total organic carbon (TOC) contents of filtrates were measured by a total organic carbon analyzer (TOC-VCPH, Shimadzu, Kyoto, Japan). The concentrations of the MB were determined by UV-vis spectrophotometer (UV-1800, Shimadzu, Kyoto, Japan).

## 3. Results

### 3.1. Preparation and Wetting Behaviors of Membranes

**Figure 1:**
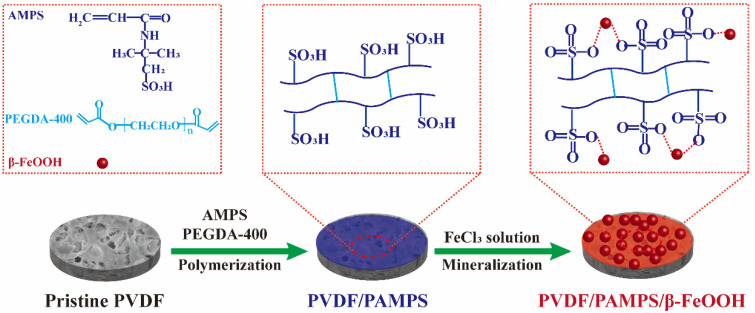
Schematic illustration of the preparation of PVDF/PAMPS/β-FeOOH membranes.

The preparation process of PVDF/PAMPS/β-FeOOH membranes is shown in Figure 1. PVDF membrane prewetted by ethanol as substrates was first immersed in a mixed solution containing AMPS monomer, PEGDA-400 as the chemical cross-linker, APS as the initiator, and PAM as the thickener. The resultant PAMPS hydrogel coated PVDF membrane, denoted as PVDF/PAMPS, was obtained via in situ free radical polymerization. Notably, numerous sulfonic groups were generated on the surface of PVDF/PAMPS membrane, providing abundant coordination sites for metal ions. Ultimately, biomimetic mineralization was utilized to construct β-FeOOH nanorods on PVDF/PAMPS membrane to enhance surface roughness and photocatalytic activity toward organic pollutants simultaneously. In the initial stage of mineralization, PVDF/PAMPS membrane could rapidly adsorbed Fe^3+^ owing to the abundant sulfonic groups on the surface. Obviously, Fe^3+^ easily hydrolyzed in the aqueous system and β-FeOOH nanorods eventually mineralized on the membrane surface. Lastly, we note that hydrogel/β-FeOOH-coated PVDF membranes (denoted as PVDF/PAMPS/β-FeOOH) can be manufactured environmentally and inexpensively via an aqueous route, making it potentially feasible for large-scale use. 

**Figure 2:**
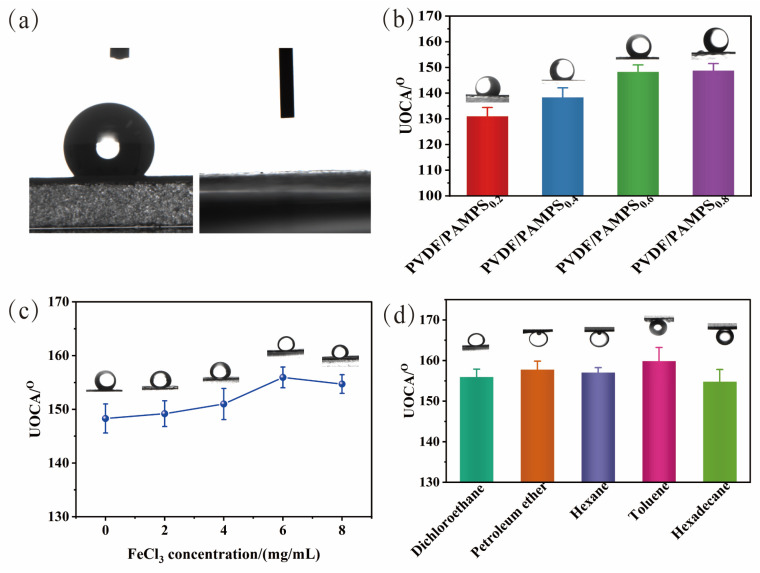
Wetting behaviors of the PVDF and modified PVDF membranes. (**a**) WCA of PVDF membranes (**left**) and PVDF/PAMPS/β-FeOOH membranes (**right**), (**b**) UOCA of PVDF/PAMPS membranes, (**c**) UOCA of PVDF/PAMPS/β-FeOOH membranes as a function of FeCl_3_ concentration, (**d**) UOCA of PVDF/PAMPS_0.6_/β-FeOOH_6_ membrane for various oils.

To further investigate the wetting behavior of the resulting PVDF/PAMPS/β-FeOOH membrane, contact angle (CA) measurements were conducted and the corresponding results were shown in Figure 2. As shown in Figure 2a, the pristine PVDF membrane show a WCA of 133.2 ± 1.8°. After modification of PAMPS hydrogel and β-FeOOH, the WCAs were 0° for all the modified PVDF membranes owing to abundant inherent hydroxyl groups, indicating a transition from hydrophobic state to superhydrophilic one. The UOCA of the as-prepared membrane was further quantitatively measured using a 4 μL dichloroethane oil droplet. As shown in Figure 2b, it can clearly be observed that UOCA of PVDF/PAMPS_0.2_ was 131.0 ± 3.4° as the hydrophilicity of membrane improved by introducing sulfonic groups on the surface. The UOCA of membranes increased to 148.3 ± 2.7° as the weight ratio of AMPS/H_2_O increased until 0.6. Furthermore, by introducing β-FeOOH nanorods on the surface of PVDF/PAMPS membranes, UOCA of the as-obtained PVDF/PAMPS_0.6_/β-FeOOH membranes just raised up to 149.2 ± 2.4° when the FeCl_3_ concentration was 2 mg/mL. Since the concentration of FeCl_3_ increasing from 4 to 8 mg/mL, the UOCA of modified membranes were 151.0 ± 2.9°, 156.0 ± 1.9°, 154.7 ± 1.7°, demonstrating superior underwater superoleophobicity (Figure 2c). The results indicated that the hydrophilic β-FeOOH nanorods further enhanced the underwater superoleophobic capability of the PVDF/PAMPS/β-FeOOH membranes. Considering the optimal wettability behavior, PVDF/PAMPS_0.6_/β-FeOOH_6_ membrane prepared from 0.6 wt% AMPS and 6 mg/mL FeCl_3_ aqueous solution was taken as a representative sample in the following experiments unless otherwise noted. Additionally, UOCAs of five kinds of oils (dichloroethane, petroleum ether, hexane, toluene, and hexadecane) had been measured and were all larger than 150° (Figure 2d), demonstrating the versatility of such underwater superoleophobicity performance of PVDF/PAMPS/β-FeOOH membranes. Specially, the preparation strategy presented here is facile and eco-friendly via an aqueous route without using any toxic organic solvents.

### 3.2. Characterization of Membranes

**Figure 3:**
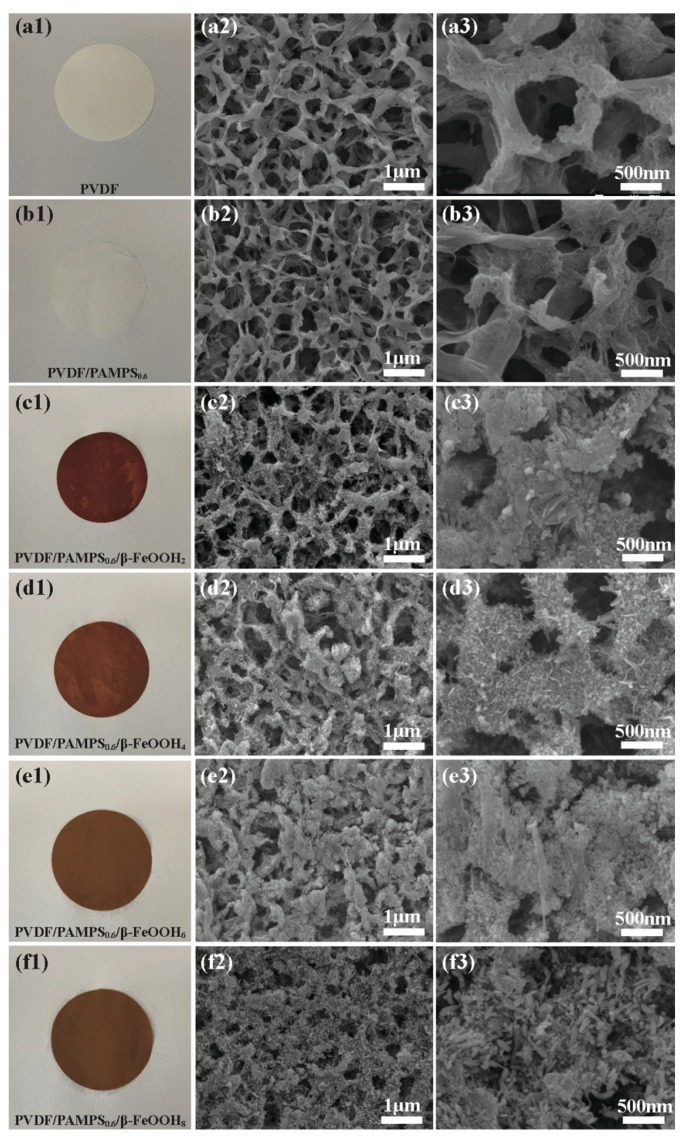
The digital photographs and SEM images of the membranes before and after modification. (**a**) pristine PVDF, (**b**) PVDF/PAMPS, and (**c**–**f**) PVDF/PAMPS/β-FeOOH membranes.

In order to reveal the microstructure evolution, we performed SEM observation of the membranes at different stages. The optical photographs and corresponding SEM images of the pristine and modified PVDF membranes are shown in Figure 3. As shown in Figure 3(a1), the original PVDF membrane was white. After modification with PAMPS hydrogel, the color showed no obvious change (Figure 3(b1)). Especially after the decoration of β-FeOOH nanorods, the white PVDF/PAMPS membrane had been changed to a brown membrane, suggesting that the mineralization reaction occurred (Figure 3(c1–f1)). The pristine PVDF membrane shows an interconnected porous structure, as shown in Figure 3(a2,a3). The structure of membrane remained unchanged when it was modified of PAMPS (Figure 3(b2,b3)). Furthermore, we investigated the effect of FeCl_3_ concentration on the density and size of in situ synthesized β-FeOOH nanorods on membrane surface by SEM, as revealed in Figure 3(c2,3–f2,3). After mineralization, abundant β-FeOOH nanorods can be observed on the surface of the resultant PVDF/PAMPS/β-FeOOH membranes. Particularly since the mass concentration of FeCl_3_ increased, the denser β-FeOOH nanorods were stuck on the surface of membrane-like cactus needle. Therefore, the micro-nanostructures were constructed by the original porous structure and β-FeOOH nanorods, which made modified PVDF membranes shown especially superhydrophilic property. When the PVDF/PAMPS/β-FeOOH membranes were immersed into water, a water layer quickly formed on the surface as a result from the superhydrophilicity and micro-nanostructure, which prevented oil wetting and kept underwater superoleophobicity [43,44].

**Figure 4:**
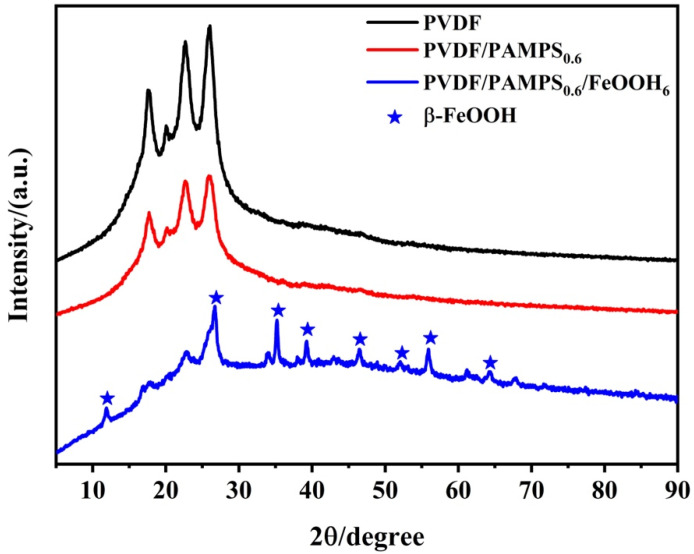
XRD patterns of the pristine PVDF, PVDF/PAMPS_0.6_ and PVDF/PAMPS_0.6_/β-FeOOH_6_ membranes.

To gain further insight into the crystal phase of the membranes, we carried out XRD measurement, as shown in Figure 4. Obviously, the pristine PVDF and PVDF/PAMPS_0.6_ membrane exhibited similar XRD pattern, suggesting that the crystal phase of the membrane had no change after modification with PAMPS hydrogel. In addition, for the XRD pattern of PVDF/PAMPS_0.6_/β-FeOOH_6_ membrane, besides the characteristic peaks of the pristine PVDF membrane, several peaks at 12.0, 26.7, 35.4, 38.1, 39.4, 46.5, 52.0, 56.2, and 64.0° corresponding to the (110), (400), (211), (420), (301), (411), (600), (521), and (541) could be identified as β-FeOOH (JCPDS No. 34–1266) [39,45]. Thus, the results confirmed the successful formation of β-FeOOH nanorods on the surface of PVDF membrane after in situ mineralization. 

**Figure 5:**
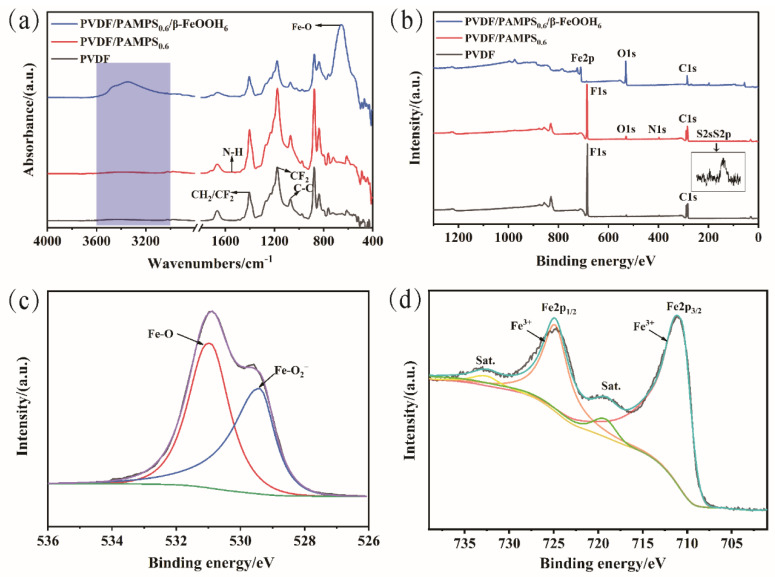
Surface chemistry of PVDF membranes before and after modification. (**a**) ATR-IR spectra and (**b**) XPS spectra of the pristine PVDF, PVDF/PAMPS_0.6_ and PVDF/PAMPS_0.6_/β-FeOOH_6_ membranes, respectively. (**c**) O 1s, and (**d**) Fe 2p XPS spectra of PVDF/PAMPS_0.6_/β-FeOOH_6_ membrane.

To further reveal the functional groups and chemical compositions of samples at different stages, ATR-IR and XPS have been utilized to characterize the membranes (Figure 5). As for the pristine PVDF membrane, a typical peek at 1403 cm^−1^ corresponding to CH_2_ and CF_2_ deformation vibration can be detected. Additionally, the absorption peaks at 1182 cm^−1^ and 1072 cm^−1^ are attributed to CF_2_ and C-C stretching vibrations, respectively [46]. After coating with PAMPS hydrogel, the emerged new peak at 1550 cm^−1^ is attributed to the bending vibration of N-H bonds [47]. Additionally, the absorption band around 1300–1500 cm^−1^ is assigned to the overlapping peaks of S=O and CH_2_/CF_2_ groups [48]. For PVDF/PAMPS_0.6_/β-FeOOH_6_ membrane, a new characteristic peak at 657 cm^−1^ corresponding to Fe-O vibrational mode in β-FeOOH nanorods can be clearly observed, confirming the successful decoration of β-FeOOH nanorods on the membrane surface [39]. Moreover, a wide absorption peak ranging from 3000 to 3600 cm^−1^ is attributed to -OH stretching vibration originating from β-FeOOH [37,49]. The chemical compositions of membranes were further investigated by XPS, and the corresponding spectra are presented in Figure 5b–d. As depicted in Figure 5b, the peaks of C 1s and F 1s can be obviously observed in the XPS spectrum of the pristine PVDF membrane, suggesting that the commercial PVDF membrane possesses hydrophobic property. For PVDF/PAMPS_0.6_ membranes, new peaks of N 1s, O 1s, S 2s, and S 2p can be detected, demonstrating the successful formation of PAMPS hydrogel on the pristine PVDF membrane surface. In contrast to the PVDF/PAMPS_0.6_ membrane, the PVDF/PAMPS_0.6_/β-FeOOH_6_ membrane exhibited a new signal of Fe 2p, and the peak intensity of O 1s on the resultant membrane was significantly enhanced. The corresponding content of elements increased from 0% to 17.01% for Fe element and from 3.59% to 44.84% for O element, respectively. As shown in Figure 5c, there are two peaks at 531.2 eV and 529.6 eV for the O 1s spectrum of PVDF/PAMPS_0.6_/β-FeOOH_6_ membrane corresponding to Fe-O and Fe-O_2_^−^, respectively [50]. In the Fe 2p spectrum of the PVDF/PAMPS_0.6_/β-FeOOH_6_ membrane (Figure 5d), four peaks at 710.7 eV, 719.2 eV, 724.8 eV, and 732.5 eV are attributed to Fe 2p_3/2_, satellite peak, Fe 2p_1/2_, and satellite peak, respectively [51]. Overall, all of these results demonstrated that PVDF/PAMPS/β-FeOOH membrane has been successfully fabricated via an aqueous route.

### 3.3. Separation of Oil-In-Water Emulsions

**Figure 6:**
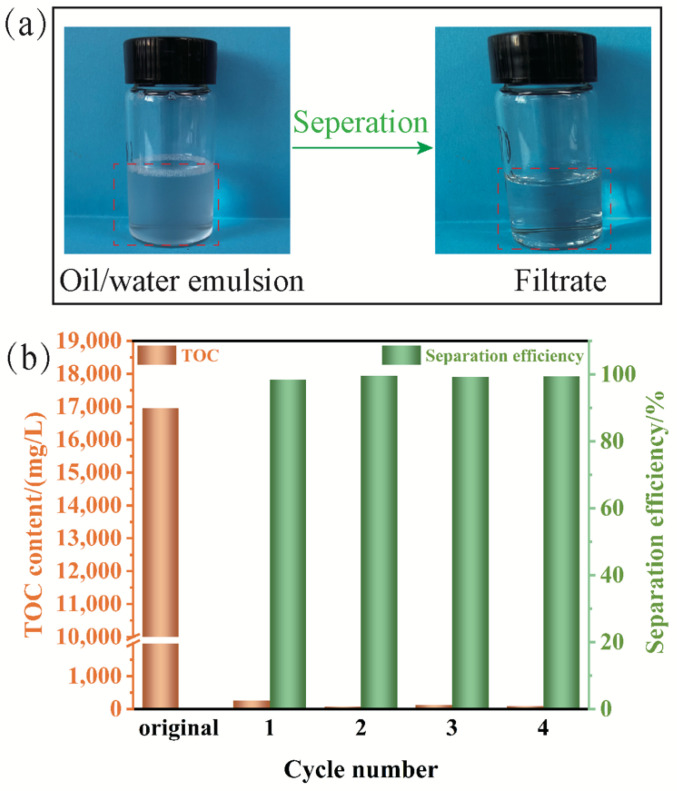
Separation performance of PVDF/PAMPS_0.6_/β-FeOOH_6_ membrane. (**a**) Photographs of hexadecane-in-water emulsion before and after separation. (**b**) The TOC contents of the filtrates and the corresponding separation efficiencies for cyclical separation.

To evaluate the separation performance of PVDF/PAMPS_0.6_/β-FeOOH_6_ membrane, SDS-stabilized hexadecane-in-water emulsion was utilized to test. As clearly shown in Figure 6a, the translucent hexadecane-in-water emulsion became transparent after the PVDF/PAMPS_0.6_/β-FeOOH_6_ membrane separation. Furthermore, after four cycles of oil/water separation tests, the TOC contents of the as-obtained filtrate decreased from 16,958.13 to 88.99 mg/L, as shown in Figure 6b. Meanwhile, the corresponding separation efficiency of PVDF/PAMPS_0.6_/β-FeOOH_6_ membrane was above 99.0%. That means PVDF/PAMPS_0.6_/β-FeOOH_6_ membrane could effectively remove the oils in water for oil-in-water emulsion.

### 3.4. Photo-Fenton Activity and Water Purification

**Figure 7:**
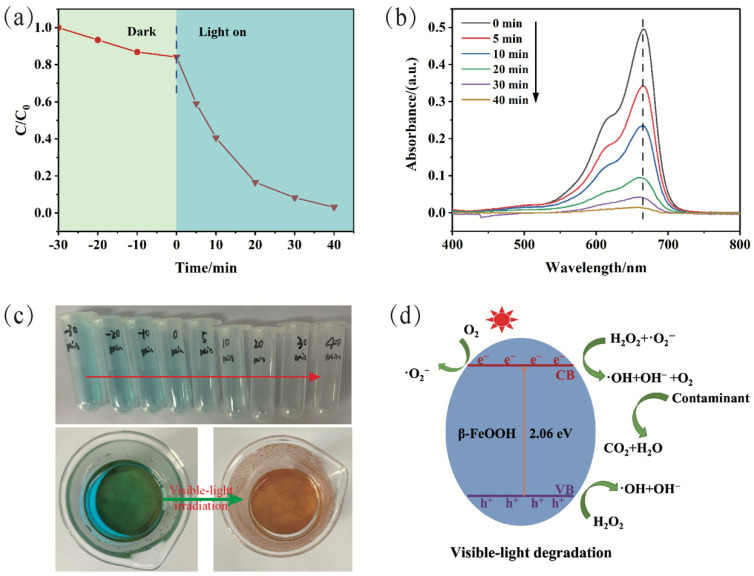
Photo-Fenton catalytic capability of PVDF/PAMPS_0.6_/β-FeOOH_6_ membrane. (**a**) Photocatalytic activity curve and (**b**) UV-vis absorption spectra of MB at different time intervals through photocatalytic degradation. (**c**) Photographs of MB solutions before and after photo-Fenton catalysis at different illumination times. (**d**) Removal mechanism of organic dyes for PVDF/PAMPS_0.6_/β-FeOOH_6_ membrane.

Benefiting from the decoration of β-FeOOH nanorods, which is a type of semiconductor with a 2.06 eV bandgap, the PVDF/PAMPS_0.6_/β-FeOOH_6_ membrane can be used as a visible-light-derived photocatalyst membrane for water purification. In our work, taking methylene blue (MB) as a model organic contaminant, we next studied the photocatalytic performance of the PVDF/PAMPS_0.6_/β-FeOOH_6_ membrane, and the corresponding results are shown in Figure 7. Figure 7a shows the relative concentrations of MB solutions at different time intervals under visible light. Obviously, MB could be completely degraded within 40 min, indicating outstanding photo-Fenton catalytic capability. Besides, the absorbance at 665 nm of MB aqueous solution weakened gradually and almost disappeared after 40 min of visible light irradiation (Figure 7b). The above results indicated that the MB molecules possessing conjugated chromophore structure were rapidly destroyed and degraded into small molecules under visible-light irradiation, as demonstrated by other previous work [38,52,53]. Moreover, upon irradiation with visible light, the blue color of original MB solution gradually became transparent (Figure 7c), which confirmed that PVDF/PAMPS_0.6_/β-FeOOH_6_ membrane has outstanding photo-Fenton catalytic activity. Based on the above results, Figure 7d reveals a general view of possible mechanisms of photo-Fenton catalytic activity, and the degradation process for organic contaminants (C) can be described in the following equations [40,45]:

β-FeOOH + hυ → e^−^ + h^+^ (2)

e^-^ + O_2_ → •O_2_^−^ (3)

•O_2_^−^ + H_2_O_2_ → •OH + OH^−^ + O_2_ (4)

h^+^ + H_2_O_2_ → •OH + OH^−^ (5)

•OH + C → H_2_O + CO_2_ (6)

In detail, β-FeOOH nanorods can generate electron (e^−^)-hole (h^+^) pairs under visible light irradiation. Subsequently, excited electrons (e^−^) from the value band (VB) transfer to the conduction band (CB) of β-FeOOH. Afterwards, adsorbed O_2_ can react with e^−^ to form superoxide radicals (•O_2_^−^) on the β-FeOOH surface. Then, abundant •O_2_^−^ can react with H_2_O_2_ to further generate hydroxyl radicals (•OH). Moreover, h^+^ on the VB of β-FeOOH can also facilely react with H_2_O_2_ to produce more •OH. During the whole water purification process, •OH and •O_2_^−^ would directly oxidize the adsorbed organic molecules on the PVDF/PAMPS_0.6_/β-FeOOH_6_ membrane into small nontoxic byproducts (CO_2_, H_2_O) owing to their high oxidative capacity. Consequently, we believe that the as-obtained PVDF/PAMPS_0.6_/β-FeOOH_6_ membrane can be considered a promising material to solve wastewater pollution in practical applications.

## 4. Conclusions

In summary, we have presented a simple, eco-friendly, and aqueous route for engineering hydrogel/β-FeOOH-coated PVDF membranes with superhydrophilicity and underwater superoleophobicity, which were fabricated via a facile free radical polymerization and mineralization process. The excellent superhydrophilic/underwater superoleophobic property of PVDF/PAMPS/β-FeOOH membrane originated from the synergetic effects of hierarchical structures and high surface energy composition. Moreover, the as-prepared membrane achieved outstanding separation efficiency for oil/water separation and could be reused with no loss of separation capability. Additionally, benefitting from the β-FeOOH nanorods anchored on the surface, the resulting membrane could effectively remove soluble dyes from wastewater by visible-light degradation, which meets the requirement for treating the real wastewater on a mass scale. We believe that this aqueous strategy will open an avenue to prepare multifunctional PVDF composite membranes for applications in oil/water separation and wastewater purification.

## Data Availability

No new data were created or analyzed in this study. Data sharing is not applicable to this article.
